# Pre-emptive Quality Control of a Misfolded Membrane Protein by Ribosome-Driven Effects

**DOI:** 10.1016/j.cub.2019.12.060

**Published:** 2020-03-09

**Authors:** Ramya Lakshminarayan, Ben P. Phillips, Imogen L. Binnian, Natalia Gomez-Navarro, Norberto Escudero-Urquijo, Alan J. Warren, Elizabeth A. Miller

**Affiliations:** 1 Department of Biological Sciences, Columbia University, 1212 Amsterdam Ave., New York, NY 10027, USA; 2 Medical Research Council Laboratory of Molecular Biology, Cambridge Biomedical Campus, Francis Crick Ave., Cambridge CB2 0QH, UK; 3 Cambridge Institute for Medical Research, The Keith Peters Building, University of Cambridge, Hills Road, Cambridge CB2 0XY, UK; 4 Department of Haematology, The Keith Peters Building, University of Cambridge, Hills Road, Cambridge CB2 0XY, UK; 5 Wellcome Trust – Medical Research Council Stem Cell Institute, University of Cambridge, Puddicomb Way, Cambridge CB2 0AW, UK

**Keywords:** protein quality control, ribosome-associated quality control, protein folding, endoplasmic reticulum, translational tuning

## Abstract

Cells possess multiple mechanisms that protect against the accumulation of toxic aggregation-prone proteins. Here, we identify a pre-emptive pathway that reduces synthesis of membrane proteins that have failed to properly assemble in the endoplasmic reticulum (ER). We show that loss of the ER membrane complex (EMC) or mutation of the Sec61 translocon causes reduced synthesis of misfolded forms of the yeast ABC transporter Yor1. Synthesis defects are rescued by various ribosomal mutations, as well as by reducing cellular ribosome abundance. Genetic and biochemical evidence point to a ribosome-associated quality-control pathway triggered by ribosome collisions when membrane domain insertion and/or folding fails. In support of this model, translation initiation also contributes to synthesis defects, likely by modulating ribosome abundance on the message. Examination of translation efficiency across the yeast membrane proteome revealed that polytopic membrane proteins have relatively low ribosome abundance, providing evidence for translational tuning to balance protein synthesis and folding. We propose that by modulating translation rates of poorly folded proteins, cells can pre-emptively protect themselves from potentially toxic aberrant transmembrane proteins.

## Highlights

• Yor1-ΔF shows synthesis defects when Sec61 or EMC function is compromised
• Synthesis defects are suppressed by ribosomal and RQC mutants
• Translation initiation is a second point of potential regulation
• Pre-emptive quality control on the ER might protect the cell from aberrant proteins

## Graphical Abstract

**Figure:**
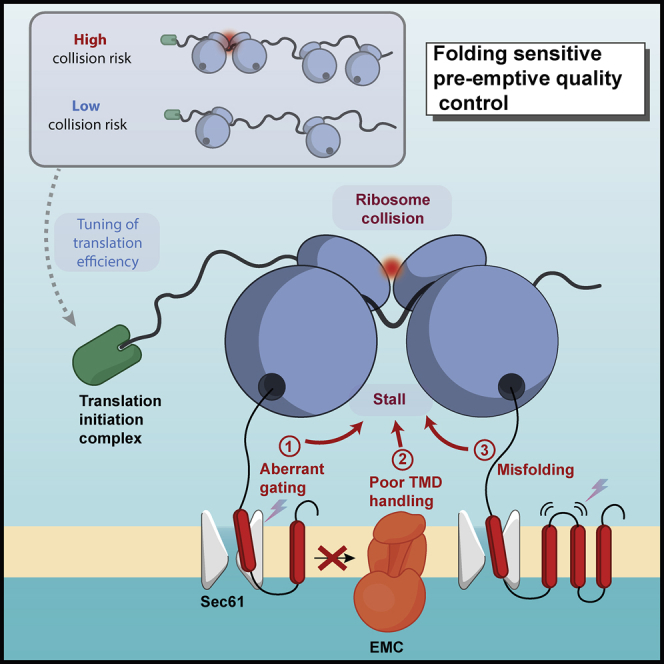

## Teaser

Polytopic membrane proteins can be difficult to fold within the constraints of the lipid bilayer. Lakshminarayan, Phillips, and colleagues identify a pre-emptive quality-control pathway that uses ribosome-associated quality-control machinery to arrest translation of problematic proteins on the endoplasmic reticulum.

## Introduction

Integral membrane proteins employ multiple machineries to facilitate correct targeting and membrane insertion [1]. The signal recognition particle binds the initial transmembrane domain (TMD) on the ribosome to deliver the nascent protein to the endoplasmic reticulum (ER) [2]. Membrane insertion is achieved via translocation channels, most commonly via the Sec61 translocon [1]. Subsequent folding of TMDs within the constraint of a planar membrane presents some unique problems. For example, TMDs often contain polar residues that comprise a hydrophilic surface in the final structure, but these residues are thermodynamically unfavorable in the context of individual TMDs within the lipid bilayer [3]. Moreover, folding of some TMDs is necessarily post-translational. ATP-binding cassette (ABC) transporters, with 12 TMDs that form a membrane-embedded channel, acquire a domain-swapped conformation whereby early-emerging TMDs pack against later TMDs [4]. Thus, folding cannot proceed in a linear manner as individual TMDs emerge into the lipid bilayer. TMD-folding requirements could explain why some ABC transporters, like human CFTR, have a poor synthesis yield [5] despite efficient co-translational folding of their cytosolic domains [6]. Finally, nascent proteins must avoid ER-associated degradation (ERAD), which targets misfolded proteins for ubiquitination and proteasomal degradation. Protective chaperones, or holdases [7], for TMD-containing proteins have not been described mechanistically but have been proposed to protect nascent membrane proteins that undergo post-translational folding [8]. Thus, despite reasonable understanding of TMD handling during targeting and insertion [1], how membrane proteins navigate this pathway to folding completion remains unclear.

The yeast ABC transporter Yor1 serves as a tractable model for biogenesis of polytopic membrane proteins. Yor1 is a pleiotropic drug pump that confers resistance to the mitochondrial poison oligomycin [9]. Yor1-ΔF_670_ is a misfolded mutant, analogous to human CFTR-ΔF_508_, which is a causative allele in cystic fibrosis patients [10]. The ΔF mutation renders the protein subject to ERAD [11], in the case of Yor1 thereby conferring oligomycin sensitivity to cells [12, 13]. We previously surveyed the yeast genome for components that influence the functional expression of Yor1-ΔF [14]. Our screen identified the yeast ER membrane complex (EMC) [15] as a biogenesis factor that promotes the functionality of Yor1-ΔF, and we demonstrated a similar role for human EMC in CFTR stability [14]. The EMC is a conserved protein complex implicated broadly in polytopic membrane-protein biogenesis [8] and mechanistically in TMD insertion at the ER [2, 16]. Deletion of Sop4, also known as Emc7, caused reduced synthesis of Yor1-ΔF in metabolic labeling experiments, consistent with an early role in ER targeting, insertion, and/or folding [14].

Here we further characterize the effects of EMC deletion on Yor1-ΔF biogenesis and describe additional mechanisms that impact synthesis of Yor1. We find that defects in Yor1 folding and membrane insertion result in reduced protein synthesis. Genetic, biochemical, and bioinformatic experiments suggest a co-translational mechanism driven by ribosome function and abundance. We propose that ribosome occupancy is an important determinant of biosynthesis of membrane proteins, with high ribosome density associated with poor outcomes, perhaps caused by ribosome collisions. Cells could thus use translational tuning to modulate membrane-protein synthesis, deploying a pre-emptive quality-control checkpoint that protects the integrity of the secretome.

## Results

**Figure 1:**
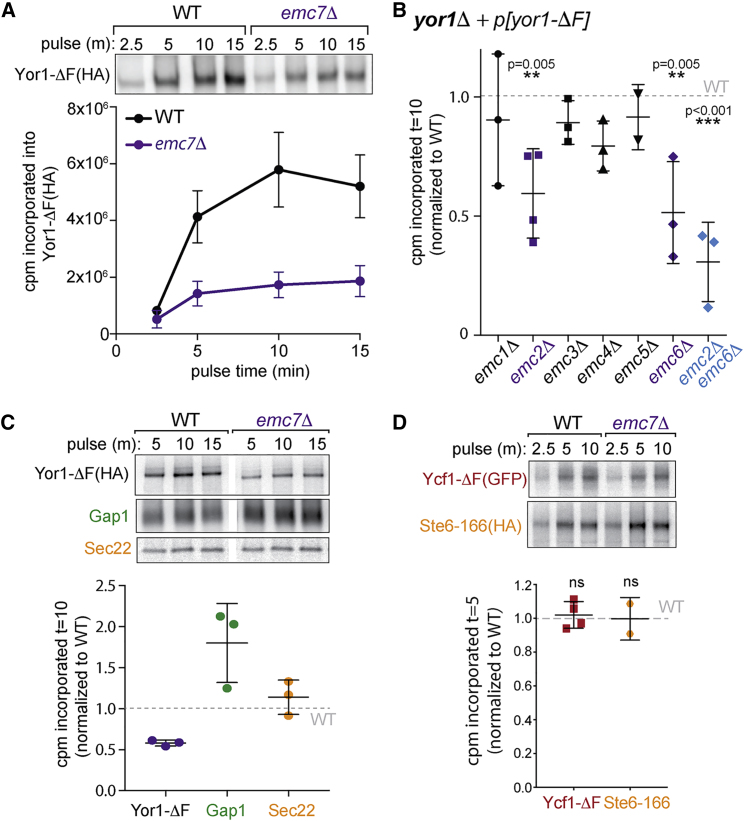
Biosynthetic Defects in Yor1-ΔF upon Loss of EMC Function (A) Yor1-ΔF(HA) was immunoprecipitated after metabolic labeling of WT and *emc7Δ* cells for the indicated times. The *emc7Δ* strain showed reduced incorporation for Yor1-ΔF(HA) over all time points. n = 22 (WT) and 16 (*emc7Δ*); error bars depict SEM. (B) Radiolabeled Yor1-ΔF(HA) was immunoprecipitated from the deletion strains indicated; loss of *EMC2* and/or *EMC6* resulted in reduced incorporation at t = 10 in relation to WT. (C) Yor1-ΔF(HA), Gap1, and Sec22 were immunoprecipitated from WT and *emc7Δ* strains after metabolic labeling for the indicated times; only Yor1-ΔF showed reduced incorporation at t = 10 in relation to WT. (D) Synthesis of misfolded Ycf1 and Ste6 was quantified in WT and *emc7Δ* strains revealing no effect of loss of Emc7. Statistical analyses used an unpaired Student’s t test; error bars depict SD (B), (C), and (D). See also Figure S1.

To explore the basis for reduced Yor1-ΔF synthesis in *emc7Δ* cells, we tested the kinetics of protein synthesis using metabolic labeling experiments. We observed a rapid plateau in ^35^S-Met/Cys incorporation into Yor1-ΔF(HA) in the *emc7Δ* strain, greatly reduced in relation to wild type (WT), that was not resolved at increasing time points (Figure 1A). Thus, loss of Emc7 impacts the earliest stages of Yor1-ΔF synthesis. Because all members of the EMC were deletion enhancers of Yor1-ΔF function [14], we tested whether each EMC mutant showed similar synthesis defects. Indeed, Yor1-ΔF synthesis was attenuated in *emc2Δ* and *emc6Δ* mutants, an effect that was exacerbated in an *emc2Δ emc6Δ* double mutant (Figure 1B). This phenotype mirrored oligomycin sensitivity conferred by EMC deletions, with *emc2Δ, emc6Δ* and *emc7Δ* the most sensitive (Figure S1A). These growth assays use low concentrations of oligomycin such that Yor1-ΔF in a wild-type cell confers modest resistance, revealing growth defects when biogenesis factors are deleted [14]. Loss of mammalian Emc2 or Emc6 destabilizes the entire EMC [2, 17], perhaps explaining their significant impact in our system.

We next tested whether Yor1-ΔF synthesis defects reflect global changes in membrane-protein biosynthesis. Synthesis of the polytopic membrane protein Gap1 and the tail-anchored protein Sec22 were not reduced in the *emc7Δ* mutant, suggesting a specific rather than universal response (Figure 1C). Similarly, two other misfolded ABC transporters, Ycf1-ΔF and Ste6-166, were synthesized normally in the *emc7Δ* strain (Figure 1D), suggesting a surprising level of client specificity. Consistent with specific translational effects, *emc7Δ* cells did not have elevated phosphorylated eIF2α even when Yor1-ΔF was expressed (Figure S1B), suggesting that the integrated stress response is not responsible for attenuated Yor1 synthesis.

**Figure 2:**
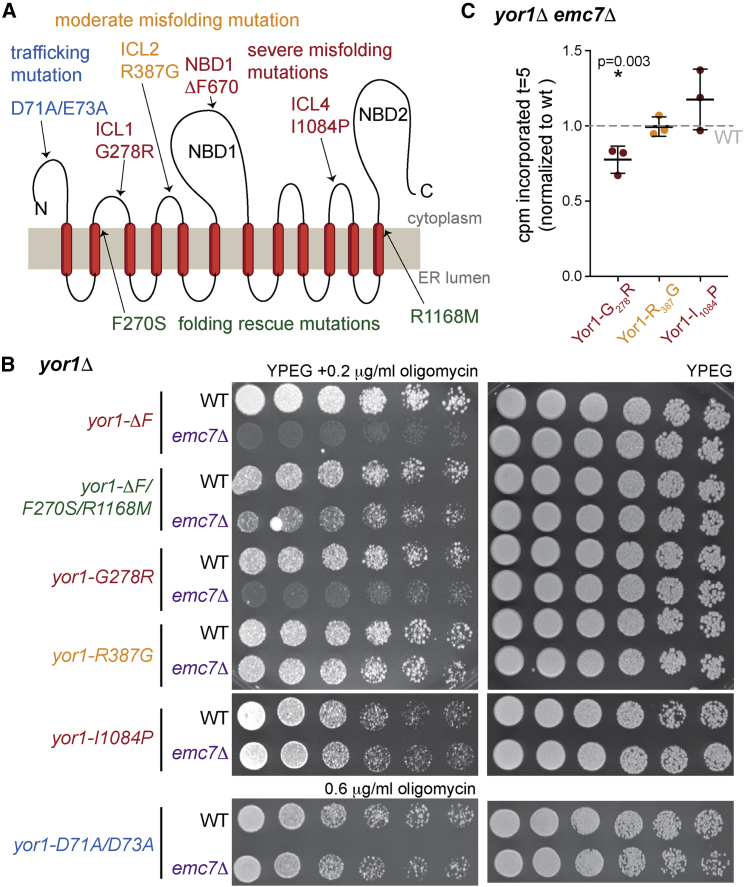
EMC-Dependent Synthesis Defects Correlate with Specific Sites of Yor1 Misfolding (A) Cartoon of Yor1, showing relevant folding and trafficking mutations. ICL, intracellular loop; NBD, nucleotide binding domain. (B) Serial dilutions of *yor1Δ* or *yor1Δ emc7Δ* strains expressing the indicated alleles of *YOR1* were spotted onto YPEG media with and without oligomycin. Emc7-associated oligomycin sensitivity correlated with misfolding defects that occurred early in the protein sequence. (C) Metabolic labeling of the indicated Yor1 alleles in wild-type or *emc7Δ* mutants was quantified at t = 5 min and normalized to WT; synthesis defects phenocopied oligomycin sensitivity. Statistical analysis was an unpaired Student’s t test; error bars depict SD.

The substrate selectivity of synthesis defects, coupled with our previous observation that loss of EMC impacts Yor1-ΔF but not wild-type Yor1 [14], suggests that specific folding defects trigger reduced biosynthesis. We investigated how distinct misfolding lesions impact EMC-dependent oligomycin sensitivity by using a panel of Yor1 alleles that affect folding and trafficking to different degrees (Figure 2A). If oligomycin sensitivity results from early quality control triggered by the combination of Yor1-ΔF misfolding and EMC deficiency, then alleles that restore folding to Yor1-ΔF or cause minimal folding defects should be unaffected by EMC loss. The ΔF mutation, in the middle of the protein, profoundly impacts packing of transmembrane helices, probably by perturbing the interface between the intracellular loop (ICL) and the nucleotide binding domain (NBD), which drives the global fold [13, 18]. Two intragenic suppressing mutations, F_270_M and S_1168_M, restore folding to Yor1-ΔF and other misfolded alleles [18]. If the folding state of the nascent protein drives early quality control, then stabilization of the fold should diminish the effects of EMC loss. Indeed, the Yor1-ΔF/F_270_S/R_1168_M mutant was less affected by loss of *EMC7* than was Yor1-ΔF (Figure 2B). Two mutations in the N-terminal region, in ICLs 1 and 2, respectively, have different degrees of misfolding; the G_278_R mutation in ICL1 is profoundly misfolded and ER retained [18], whereas R_387_G in ICL2 is a functional mutation that causes partial misfolding [19]. These alleles were differentially affected by *EMC7* deletion; oligomycin sensitivity of the ICL1 mutant was enhanced in an *emc7Δ* strain, whereas the ICL2 mutant was unaffected (Figure 2B). Finally, I_1084_P in ICL4, which yields a profoundly misfolded protein [18], was unaffected by loss of *EMC7* (Figure 2B). In the context of the folding landscape of Yor1, the late emergence of the ICL4 lesion during synthesis apparently renders the protein less dependent on EMC, suggesting EMC acts upstream of the eleventh TMD. Finally, a trafficking mutant, Yor1-D_71_A/E_73_A, which is correctly folded but fails to engage ER export machinery [13], was unaffected by *EMC7* deletion even at high oligomycin concentrations (Figure 2B). Oligomycin phenotypes corresponded to *in vivo* synthesis defects in pulse-labeling experiments; Yor1-G_278_R showed reduced synthesis in the *emc7Δ* strain, whereas Yor1-R_387_G and Yor1-I_1084_P were unaffected (Figure 2C). We conclude that the folding state of the client protein is a driver of quality control triggered by loss of EMC, and the location and severity of the misfolding lesion are important determinants. These results partially explain the client specificity we observed among ABC transporters; the Ste6 misfolding lesion occurs late in the protein, likely downstream of a putative site of EMC action. For Ycf1, the presence of numerous additional upstream TMDs prior to the first nucleotide binding domain might alter EMC positional requirements [20].

**Figure 3:**
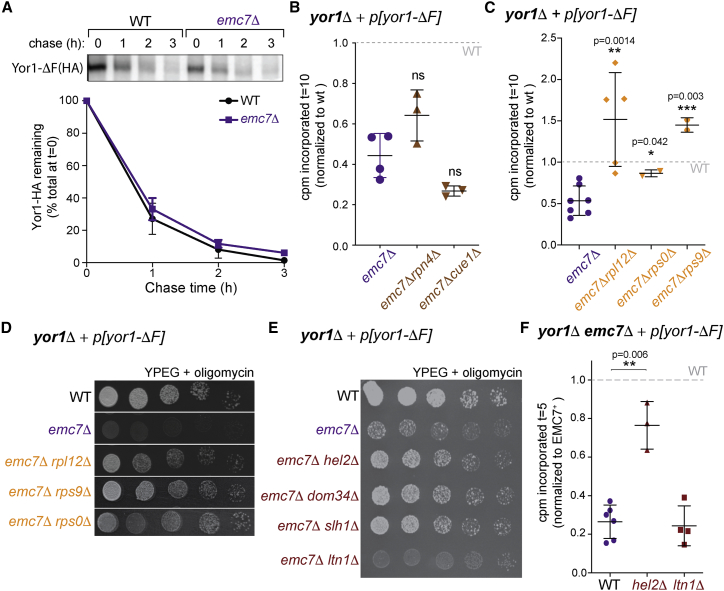
Yor1-ΔF Synthesis Defects Reflect Ribosome-Associated Events (A) Degradation of Yor1-ΔF(HA) was similar in WT and *emc7Δ* cells after a 10 min pulse and chase times indicated; n = 2. (B) Deletion of ERAD factors *RPN4* and *CUE1* in the *emc7Δ* background did not restore labeling of Yor1-ΔF(HA) in relation to WT at t = 10 min. (C) Deletion of the ribosomal proteins indicated reversed the effect of *EMC7* deletion on metabolic labeling of Yor1-ΔF(HA), normalized to WT at t = 10 min. (D) Serial dilutions of the indicated strains expressing Yor1-ΔF were spotted onto YPEG media supplemented with oligomycin. The *emc7Δ* strain showed enhanced oligomycin sensitivity; additional deletion of ribosomal proteins reversed this effect. (E) Oligomycin resistance of the indicated strains expressing Yor1-ΔF was assessed by serial dilution; deletion of *HEL2*, *DOM34*, and *SLH1* restored partial oligomycin resistance, whereas deletion of *LTN1* had no effect. (F) Yor1-ΔF(HA) synthesis was measured in the indicated strains and normalized to WT. Deletion of *HEL2* restored Yor1-ΔF(HA) synthesis, whereas deletion of *LTN1* had no effect. Statistical tests were unpaired Student’s t test; error bars depict SD. See also Figures S2, S3, and S4.

Having identified folding specificity in Emc7-dependent biogenesis defects, we sought to understand the fate of misfolded Yor1 in the absence of EMC function. Our labeling assays use C-terminally tagged Yor1-ΔF; antibodies raised against the N-terminal cytosolic domain, and attempts to epitope tag Yor1 at the N terminus, failed in immunoprecipitation (IP) experiments; thus, translation and degradation intermediates are not detectable. We first confirmed that protein turnover was unaffected by loss of *EMC7* (Figure 3A) [14], consistent with a biogenesis defect rather than degradation after complete synthesis. We then asked whether ERAD disposes of aberrant Yor1-ΔF during the time frame of our experiments. We chose two ERAD-related mutants, *rpn4Δ* and *cue1Δ*, that were moderate deletion suppressors and stabilized Yor1-ΔF [14]. We tested Yor1-ΔF pulse-labeling in *emc7Δ rpn4Δ* and *emc7Δ cue1Δ* mutants. Although deletion of neither *RPN4* nor *CUE1* reversed *emc7Δ-*associated Yor1-ΔF synthesis defects (Figure 3B), *rpn4Δ* and *cue1Δ* single mutants also showed reduced Yor1-ΔF synthesis at early time points, albeit to a lesser extent (Figure S2A), rendering the data difficult to interpret. Attempts to inhibit ERAD by chemical means were unsuccessful because of small-molecule induction of Yor1 transcription (data not shown).

**Figure 4:**
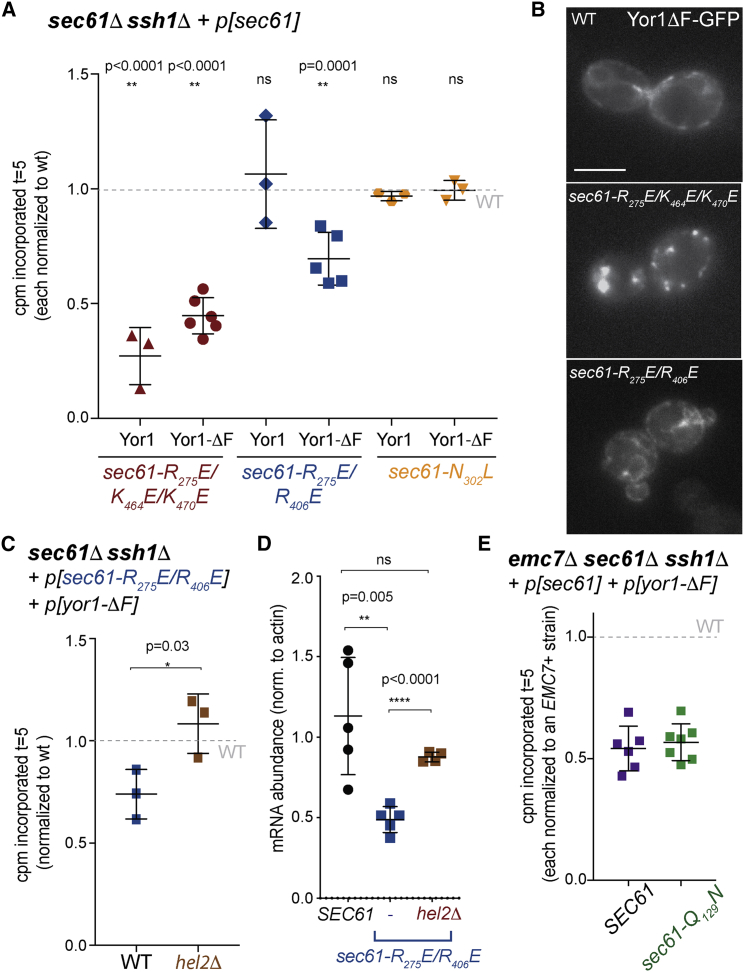
Sec61 Dysfunction Phenocopies *EMC7* Deletion to Trigger Biosynthetic Defects (A) Metabolic labeling experiments revealed synthesis defects for both WT Yor1 and Yor1-ΔF in an ER-targeting mutant, *sec61-R*_*275*_*E/K*_*464*_*E/K*_*470*_*E*, whereas a TMD-gating mutant, *sec61-R*_*275*_*E/R*_*406*_*E*, caused reduced synthesis only of Yor1-ΔF(HA), and a post-translational mutant, *sec61-N*_*302*_*L*, was unaffected. Labeling was quantified at t = 5 and normalized to a *SEC61*^+^ strain. (B) Fluorescence microscopy of WT and mutant cells expressing Yor1-ΔF-GFP revealed ER localization in WT and *sec61-R*_*275*_*E/R*_*406*_*E* cells but punctate accumulation in the *sec61-R*_*275*_*E/K*_*464*_*E/K*_*470*_*E* mutant. (C) Deletion of *HEL2* restored Yor1-ΔF(HA) synthesis in the *sec61-R*_*275*_*E/R*_*406*_*E* strain. (D) Steady-state levels of Yor1-ΔF(HA) mRNA in the strains indicated were quantified by qPCR and normalized to that of actin with a standard curve. (E) Yor1-ΔF synthesis was measured in an *emc7Δ sec61Δ ssh1Δ* strain expressing either *SEC61* or *sec61- Q*_*129*_*N*, a permissive gating mutant, which did not reverse the synthesis defects associated with loss of Emc7. Statistical tests were unpaired Student’s t test and reflect the difference between the strains indicated and a WT strain; error bars depict SD.

To probe whether EMC loss exacerbates folding defects that might trigger ERAD, we tested the folding state of Yor1-ΔF with limited proteolysis [13]. Protease susceptibility of Yor1-ΔF was unchanged between WT and *emc7Δ* cells (Figure S2B), suggesting no differences in global fold of the nascent protein. Furthermore, labeled cell lysates fractionated into soluble and insoluble fractions showed no difference between WT and *emc7Δ* strains (Figure S2C), suggesting that protein aggregation is not responsible for the observed phenotypes. Similarly, Yor1-ΔF-GFP did not accumulate in large foci (Figure S2D; compare with Figure 4B, middle panel), demonstrating that Yor1-ΔF is unlikely to form insoluble aggregates that are refractory to IP. These data collectively argue that global folding of Yor1-ΔF is not altered in the absence of Emc7.

Although unable to definitively exclude contributions from ERAD, we note that the time frame of the defects that we observe is more consistent with co-translational events rather than with a post-translational process. To further explore potential co-translational regulators, we returned to our original screen, finding significant enrichment for proteins involved in cytoplasmic translation, nonsense-mediated mRNA decay, and ribosome assembly as suppressors of oligomycin sensitivity associated with Yor1-ΔF (Figure S3). We validated a subset of ribosomal mutants for their ability to reverse the effects of Emc7 loss, observing robust rescue of Yor1-ΔF defects in both pulse labeling (Figure 3C) and phenotypic assays (Figure 3D). Ribosomal mutants can impact multiple aspects of protein biogenesis, including ribosome assembly, translation initiation and elongation, and ribosome-associated quality control (RQC). Of the mutants that we tested, Rpl12 likely improves the folding yield of Yor1-ΔF at least in part by reducing elongation rates, as has been demonstrated for human CFTR [21, 22]. In contrast, Rps9 contributes to an interface between collided ribosomes [23], after ribosome stalls. The interface creates a platform for ribosome ubiquitination and subsequent engagement of mRNA decay and RQC pathways [24, 25, 26]. In the context of Yor1-ΔF biogenesis, absence of Rps9 might reduce the efficiency of ribosome ubiquitination and thereby permit time for stall resolution. Finally, Rps0 participates in translation initiation [24] [25], defects in which would reduce ribosome occupancy along an mRNA and thus reduce the chance of ribosome collisions. Moreover, Rps0 also contributes to ribosomal small subunit maturation [26], which could also influence total ribosome abundance. Therefore, ribosome function, abundance, and translational dynamics seem to be important factors in Yor1-ΔF biogenesis.

We further explored the model of collision-driven quality control by testing cytoplasmic RQC mutants for effects on Yor1-ΔF synthesis [27]. Deletion of genes upstream of the committed step in RQC rescued the *emc7Δ* oligomycin phenotype, whereas deletion of the downstream E3 ligase Ltn1 had minimal effect (Figure 3E). Lack of rescue by *ltn1Δ* is consistent with its late role in degradation of the truncated nascent chain after ribosome splitting [27], at which point protein synthesis to completion of translation is no longer possible. In contrast, abrogation of factors that act prior to ribosome splitting (e.g., Hel2, Dom34, and Slh1 [28, 29]) can permit stalls to be overcome and protein synthesis to progress [30]. Indeed, in pulse-labeling experiments in *emc7Δ hel2Δ* and *emc7Δ ltn1Δ* strains, we see robust rescue in the *hel2Δ* background but not in the *ltn1Δ* condition (Figure 3F). Together, these findings are consistent with a role for RQC when Yor1-ΔF fails to properly integrate or fold. Whether all features of RQC, including C-terminal Ala- and Thr-extension (CAT-tailing) of the nascent chain [31], are fulfilled in this system remains to be tested. Moreover, we note that some of the rescue effects we observe are modest in relation to the robust rescue observed with ribosomal mutants, suggestive of multiple quality-control outcomes.

RQC components that drive ribosome splitting and recycling also participate in mRNA surveillance, including nonsense-mediated decay and no-go decay [32]. This functional overlap, combined with genetic effects of loss of the Ski complex, which mediates mRNA degradation (Figure S3), led us to check whether Yor1 mRNA was reduced in conditions where EMC loss triggers RQC. We measured steady-state levels of mRNA for different Yor1 alleles and found only a modest reduction in mRNA levels for the Yor1-G_278_R (ICL1) mutant in *emc7Δ* cells (Figure S4). Given genetic evidence for involvement of the SKI complex in Yor1-ΔF function (Figure S3; [14]), the lack of mRNA degradation is somewhat surprising but highlights that the synthesis defects we observe are predominantly influenced by co-translational events.

Because the EMC has recently been defined as a membrane-domain insertase [2, 16], we next tested whether defects in canonical translocation phenocopy loss of EMC and trigger RQC. We examined Yor1 biogenesis in Sec61 mutants that are defective in ER targeting and insertion. We tested three Sec61 gating mutants with a *sec61Δ ssh1Δ* double-mutant background to avoid redundant handling by alternative translocons [33, 34]. The *sec61-R*_*275E*_*/K*_*464*_*E/K*_*470*_*E* mutant that is defective for co-translational protein insertion and ribosome binding [33] showed decreased synthesis of both WT Yor1 and Yor1-ΔF (Figure 4A). This defect likely stems from initial targeting failure because Yor1-ΔF-GFP accumulated in large intracellular puncta that probably correspond to insoluble aggregates (Figure 4B). Conversely, *sec61-R*_*275*_*E/R*_*406*_*E*, which is also impaired in co-translational targeting [33], showed selective synthesis defects for Yor1-ΔF; WT Yor1 was unaffected (Figure 4A). In this mutant, Yor1-ΔF showed a normal ER distribution, arguing against formation of insoluble aggregates (Figure 4B). Finally, *sec61-N*_*302*_*L*, which selectively impacts post-translational protein insertion [34], showed no synthesis defects (Figure 4A).

We next tested whether Sec61 dysfunction also triggers RQC and found that Yor1-ΔF synthesis defects in the *sec61- R*_*275*_*E/R*_*406*_*E* background were reversed by deletion of Hel2 (Figure 4C). Moreover, steady-state Yor1-ΔF mRNA levels were reduced in the *sec61- R*_*275*_*E/R*_*406*_*E* mutant, and this effect was reversed by deletion of *HEL2* (Figure 4D), suggesting that RQC triggered by Sec61 dysfunction also causes mRNA degradation. Given the similarities with respect to Yor1 synthesis in the *sec61- R*_*275*_*E/R*_*406*_*E* and *emc7Δ* mutants, we reasoned that more promiscuous handling of Yor1 TMDs by Sec61 might bypass the need for EMC. We therefore tested if EMC loss could be suppressed by a permissive gating mutant of Sec61, *sec61-Q129N*, which accepts poorly hydrophobic signals that are not normally recognized by the translocon [34]. This permissive condition could not replace Emc7 function in Yor1-ΔF biogenesis (Figure 4E), suggesting that EMC, like Sec61, acts early in Yor1 synthesis but is functionally distinct.

**Figure 5:**
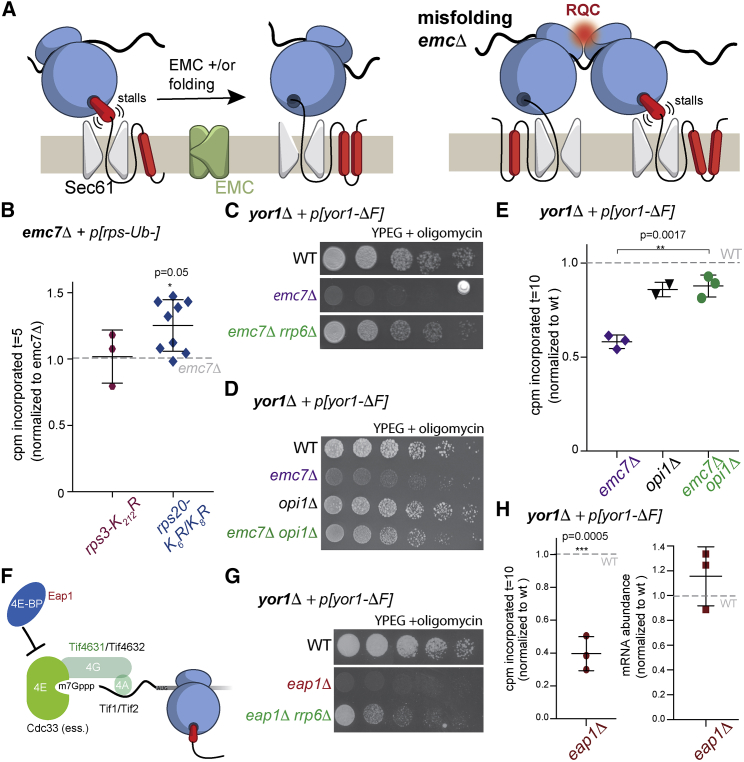
Translation Initiation as a Point of Regulation of Yor1-ΔF Biogenesis (A) Cartoon depicting ER-engaged ribosomes (blue) synthesizing a polytopic membrane protein, with hydrophobic TMDs (red) causing transient stalls (brackets). In the absence of productive folding or EMC function, stalls trigger ribosome collisions (red spot) and RQC. (B) Yor1-ΔF(HA) synthesis was measured in *emc7Δ* strains where the ribosomal subunits indicated were unable to be ubiquitinated. In this experiment, incorporation was normalized to an isogenic *emc7Δ* strain. (C) Deletion of *RRP6* rescued oligomycin sensitivity of an *emc7Δ* strain, consistent with ribosome abundance as a factor in EMC-mediated synthesis defects. (D) Deletion of *OPI1* reversed oligomycin sensitivity of the *emc7Δ* strain. (E) Metabolic labeling of Yor1-ΔF(HA) in the strains indicated was quantified at t = 10 and normalized to WT. Deletion of *OPI1* reversed the synthesis defect associated with *EMC7* deletion. (F) Cartoon of factors that regulate initiation. Cdc33 (eIF4E) is essential in yeast and thus was not accessible to genetic analysis. Tif4631 (green) was a deletion suppressor; Eap1 (eIF4E-BP) was a deletion enhancer. (G) Deletion of *EAP1* causes oligomycin sensitivity, which is reversed by additional loss of *RRP6*. (H) Metabolic labeling of the *eap1Δ* strain expressing Yor1-ΔF(HA) was quantified at t = 10 and normalized to WT (left). Steady-state mRNA levels were measured in the same strains by qPCR, with Ct values normalized to actin and the *eap1Δ* strain normalized to WT; each point represents a biological replicate comprising three technical replicates (right). Statistical analyses used an unpaired Student’s t test; error bars depict SD. See also Figures S3 and S5.

Together, our findings suggest a co-translational protein quality-control pathway that is influenced by: (1) nascent protein folding state; and (2) function of ER insertion machinery and/or TMD chaperones. We propose that hydrophobic TMDs can cause transient ribosomal stalls that are overcome either by productive folding or EMC action (Figure 5A, left panel). EMC abrogation, combined with folding defects, prolongs stalls that in turn trigger RQC, perhaps via ribosome collisions (Figure 5A, right panel). We searched for evidence of ribosome collisions by using yeast mutants that lack Hel2-mediated ubiquitination sites [29], finding modest rescue of the *emc7Δ* synthesis defect in a *rps20-K6R/K8R* mutant but not in the *rps3-K212R* background (Figure 5B). Such specificity has previously been observed for RQC of multiple translation-arrest models [29] and places Rps20 ubiquitination at a critical juncture for detection of stalls and triggering of RQC. Rps20, also known as uS10 and an essential gene in yeast, lies at the disome interface that probably corresponds to the recognition event that triggers initiation of RQC [23, 24].

Consistent with a model of collision-driven quality control for Yor1-ΔF, our original screen revealed multiple ribosome assembly factors as deletion suppressors [14]. Rrp6, a subunit of the nuclear exosome that processes ribosomal RNA, was among the strongest Yor1-ΔF suppressors (Figure S3). Deletion of *RRP6* reversed the oligomycin sensitivity of an *emc7Δ* strain (Figure 5C), suggesting that reducing ribosome abundance rescues co-translational biogenesis defects. We confirmed that the *rrp6Δ* mutant has fewer 80S ribosomes and polysomes (Figure S5A), as previously reported [35]. Because Yor1 undergoes co-translational targeting to the ER, it seems likely that potential collision events occur on ER-engaged ribosomes. We therefore tested whether ER expansion might reduce ribosome collisions and thereby suppress EMC loss. Deletion of the lipid synthesis regulator, *OPI1*, causes proliferation of ER membranes (Figure S5B) without concomitant upregulation of chaperones or translocons (Figure S5C) [36]. Indeed, in an *opi1Δ* background, oligomycin resistance was restored to an *emc7Δ* strain (Figure 5D), and synthesis of Yor1-ΔF was rescued (Figure 5E). ER expansion did not alter ERAD efficacy, as monitored by CPY^∗^ degradation (Figure S5D). Thus, expansion of the ER by approximately 50% [36] creates permissive conditions in which Yor1-ΔF biogenesis defects are reversed, perhaps by reducing encounters between ribosomes engaged in synthesis.

Given the apparent importance of ribosome abundance in Yor1-ΔF synthesis, we next examined translation initiation as a potential point of regulation. Deletion of Tif4631, eIF-4G in mammals, was a deletion suppressor [14], meaning that absence of this initiation factor improved the functionality of Yor1-ΔF (Figure S3; Figure 5F). Conversely, deletion of Eap1, a 4E-binding protein that negatively regulates initiation, was a deletion enhancer, causing reduced Yor1-ΔF function (Figure S3; Figures 5F and 5G). Both phenotypes are consistent with ribosome density on the mRNA influencing Yor1-ΔF biogenesis; reduced initiation should diminish ribosome density, whereas absence of a negative regulator of initiation would increase ribosome density and thus potential collisions. Pulse labeling of Yor1-ΔF in the *eap1Δ* strain revealed reduced synthesis in comparison to synthesis in wild-type cells, with mRNA levels unchanged (Figure 5H). Reducing ribosome abundance by deletion of *RRP6* partially reversed oligomycin sensitivity associated with loss of Eap1 (Figure 5G). The *eap1Δ rrp6Δ* double mutant had reduced ribosome abundance, in particular in the polysome fraction (Figure S5E). Thus, Eap1 seems to act as a negative regulator of Yor1 initiation; lack of Eap1 causes overloading of Yor1 mRNA with ribosomes that is detrimental, but reducing ribosome abundance reverses this effect. Active management of translation initiation, by Eap1 and other factors, could permit a dynamic response to collision events. Such translational tuning has previously been proposed to act on human CFTR to promote nascent protein folding [37].

**Figure 6:**
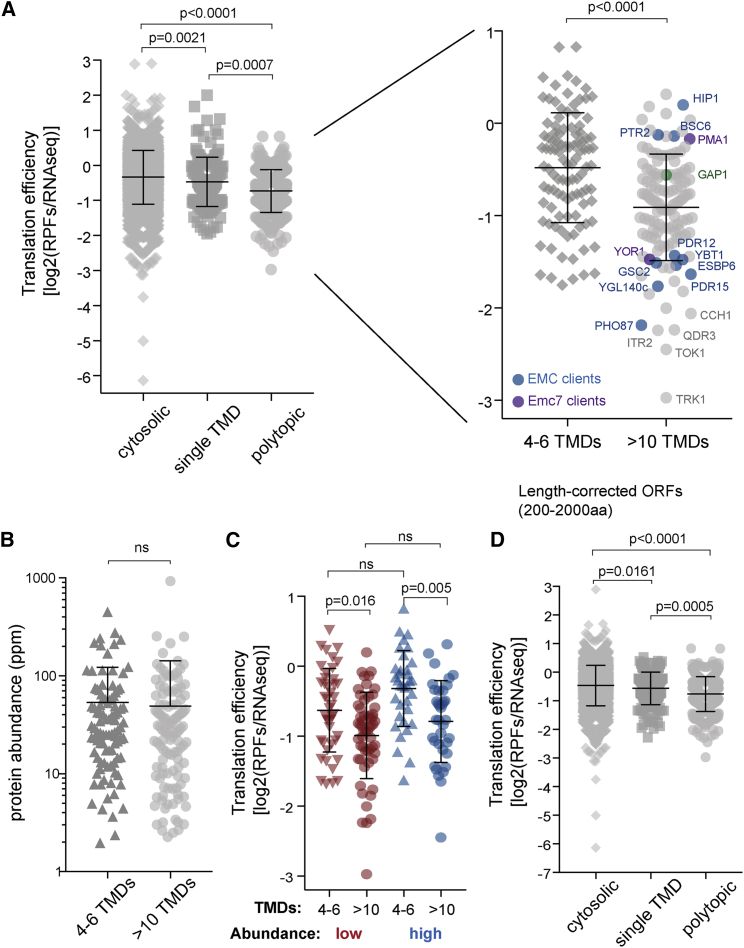
Polytopic Membrane Proteins Have Low Translation Efficiency (A) Translation efficiency (TE) was calculated from ribosome profiling data [38] and proteins separated into different categories on the basis of published classifications [39, 40]. Proteins with single TMDs and polytopic TMDs had lower TE than cytosolic proteins. Further separation of the polytopic group into few (4–6 TMDs) and many (>10 TMDs) further revealed reduced TE as TMD number increased. EMC clients within the >10 TMD set are indicated by colored circles. Select non-EMC clients (gray circles) are also indicated. (B) To rule out TE effects caused by protein abundance, we separated the polytopic group into low-, medium-, and high-abundance classes. No significant differences between protein abundance (ppm) were observed between the 4–6 and >10 TMD classes, suggesting that abundance alone cannot account for observed TE effects. (C) Separating low- and high-abundance proteins into 4–6 and >10 TMD classes; the observed reduction in TE for >10 TMD proteins was still observed. (D) To rule out length effects, analysis as described in (A) was restricted to proteins 200–2,000 amino acids long, revealing reduced TE for polytopic membrane proteins. In all cases, statistical analyses were Mann-Whitney tests, and error bars represent SD. See also Figure S6.

Our data suggested that ribosome density along the mRNA is an important factor in Yor1 synthesis yield, but the broader physiological relevance of ribosome density in multi-pass membrane-protein biogenesis remained unclear. We therefore considered whether translational tuning of membrane proteins occurs across the yeast transcriptome. We quantified translation efficiency (TE), defined as ribosome abundance along a message, from published ribosome profiling data [38] by calculating the ratio of ribosome-protected fragments to RNA-seq reads [38] and separated the data into cytosolic and secreted proteins. Secretome proteins exhibited lower TE than did cytosolic proteins, indicative of reduced ribosome occupancy (Figure S6A). Because ER targeting and/or translocation might broadly impact TE, we narrowed our focus to secretome proteins. We reasoned that polytopic membrane proteins, with complex folding trajectories, might have evolved lower ribosome occupancy to minimize the potential for problematic stalls. Comparing single TMD-containing proteins (excluding tail-anchored proteins) and polytopic proteins (defined as >4 TMDs), we observed reduced TE associated with polytopic proteins (Figure 6A). Moreover, dividing the polytopic group into 4–6 TMD and >10 TMD subgroups revealed that TE decreased with increasing TMD number (Figure 6A, right panel). Within the >10 TMD group, putative EMC clients were found with both “high” and “low” TE values (Figure 6A, annotated in blue), suggesting that EMC dependence does not universally reflect low TE. Of note, Yor1 was among the lowest TE-scoring proteins, consistent with its sensitivity to ribosome abundance. The cohort of low-TE proteins also contained non-EMC clients encompassing ion channels and solute carriers (Figure 6A, annotated in gray).

To rule out protein abundance effects as a confounding influence on TE, we extracted protein abundance data for each protein (PaxDB) and separated the polytopic proteins into low, medium, and high abundances. As expected, TE distribution broadly correlated with abundance, with low abundance proteins generally having lower TE (Figure S6B). However, comparing abundance of 4–6 TMD proteins to that of >10 TMD proteins showed no difference in the distribution of TE (Figure 6B), suggesting that abundance effects alone cannot explain the observed difference in TE. Moreover, when proteins were separated by abundance, we observed reduced TE in the >10 TMD category in comparison to the 4–6 TMD category regardless of the abundance category (Figure 6C).

ORF length can also impact ribosome occupancy, with longer ORFs having less ribosome density [41], and indeed polytopic membrane proteins tended to be longer than other classes (Figure S6C). To rule out length effects on TE, we compared the TE for cytosolic, single-pass, and polytopic transmembrane (TM) proteins for proteins between 200 and 2,000 amino acids. This size window was chosen because both single-pass and polytopic protein groups had similar frequency distributions in this range (Figure S6D). Length-corrected proteins still showed reduced TE, both for single-pass TM proteins in relation to cytosolic proteins and for polytopic proteins (Figure 6D). To ensure that comparisons between groups were meaningful, we plotted effect size for both whole proteome and length-corrected analyses (Figure S6E) and observed that restricting the analysis to a narrow length window reduced the effect size for cytosolic comparisons, but for the critical single-pass TM proteins versus polytopic membrane-protein groups, the differential effect was comparable, lending confidence to our analysis. In light of our genetic, biochemical, and bioinformatic data, we propose that low TE for multi-pass polytopic membrane proteins reflects evolutionary pressure to slow translation initiation and/or elongation to permit correct targeting, TMD insertion, and TMD folding [42].

## Discussion

Protein misfolding in the ER has long been recognized as an important point of cellular quality control, enacted at a systems level by the unfolded protein response (UPR) and through the direct actions of ERAD machinery. Here, we describe an additional layer of quality control that acts early during protein synthesis to prevent the accumulation of aberrant proteins. In the case of Yor1, we find that this pre-emptive quality control is exacerbated by deletion of the EMC, a TMD insertase [2, 16], which might also function as a chaperone [8]. Moreover, specific defects in the canonical translocon, Sec61, also confer reduced Yor1 synthesis, indicative of pre-emptive quality control. Our data don’t speak to the molecular function of the EMC, a conserved complex in eukaryotes [15, 43, 44], implicated in diverse cellular functions including lipid transfer between ER and mitochondria [45], viral intoxication [46], membrane-protein insertion [2, 16], and membrane-protein folding [8, 47]. However, our findings, which show that loss of EMC phenocopies specific dysfunction of Sec61, are consistent with a role for EMC in TMD handling at early stages of synthesis. Importantly, the topology of Yor1 is distinct from that of GPCRs, where the mammalian EMC establishes the correct orientation of the first TMD [16]. It is possible that the EMC is responsible for insertion of downstream helices in Yor1, most likely after the first large cytosolic domain is translated, or for insertion of specific poorly hydrophobic TMDs.

We propose that when ER insertion and/or folding of nascent TMDs is impaired, ER-engaged ribosomes become stalled, triggering downstream quality-control pathways. Recent structural data on ribosomes stalled by a small molecule, PF846, which triggers selective translational arrest of several secretory proteins [48], suggest a possible mechanism. In PF846-stalled ribosomes, the hydrophobic nascent chain interacts with the ribosomal exit tunnel, creating a kinked structure that impairs elongation [49]. We propose that Yor1 TMDs might be prone to similar hydrophobic interactions within the exit tunnel that can impede elongation. Correct folding and/or the action of the EMC could provide a pulling force to prevent or overcome such transient stalls, analogous to mechanisms that relieve bacterial stalling signals [50]. Loss of EMC, or Sec61 dysfunction, combined with specific misfolding lesions would result in prolonged stalls that in turn trigger ribosome collisions and reduced protein synthesis. Analysis of ribosome-protected fragments along aberrant Yor1 mRNAs will be required to determine the existence and nature of ribosome stalls under different conditions.

Ribosome collisions create a specific disome interface [23, 24] that recruits ubiquitination machinery to initiate RQC. Several early-acting RQC components participate in Yor1-ΔF quality control, whereas deletion of the RQC-associated E3 ligase Ltn1 had minimal effect, perhaps indicating partial redundancy with ERAD machinery, which also had relatively modest effects. Understanding the precise, and probably diverse, roles of the ubiquitin/proteasome system in this pathway will require more specific assays than are currently available for Yor1-ΔF. The nature of the Yor1 stalls remain to be determined and could be heterogeneous, as was observed for PF846-induced stalls [49]. One puzzling aspect of the pre-emptive QC pathway that we describe is the difference in mRNA abundance depending on the trigger for synthesis defects. In the case of Sec61 dysfunction, Yor1-ΔF mRNA levels were reduced dependent on Hel2, suggesting RNA cleavage and degradation is triggered upon RQC. In contrast, we saw no evidence for mRNA degradation upon loss of EMC function. This difference could reflect the distinct functions of EMC and Sec61, or perhaps separate RQC pathways that differentially engage mRNA decay machineries.

Our observations that differences in ribosome function and abundance modulate the synthesis of Yor1-ΔF in the context of aberrant ER membrane insertion led us to consider whether cells might actively manage translation of complex membrane proteins. Indeed, previous analyses identified reduced TE associated with signal recognition particle (SRP) binding, caused by reduced codon optimality that slows elongation to promote SRP binding [51]. Our TE analysis suggests that similar effects might operate more broadly, particularly for polytopic membrane proteins that could represent a folding challenge. Whether the global reduction in TE that we observe can be explained by hotspots of reduced translation caused by poor codon optimality or by active management of translation initiation remains to be tested. Nonetheless, it seems likely that pre-emptive quality control will be important for biogenesis of many polytopic membrane proteins. Indeed, rescue of CFTR-ΔF by knockdown of mRNA degradation machinery and by reduction in translation rates is suggestive of such an effect [21, 22]. Moreover, depletion of initiation factors also rescues CFTR-ΔF, yielding increased mRNA, increased synthesis, and improved folding yield [52], suggesting that reducing ribosome abundance along the CFTR mRNA similarly improves synthesis yield. Finally, “translational tuning,” whereby ribosome effects and codon usage modulate translation rates and folding trajectories, is important for folding of the CFTR cytosolic domains [37]. Thus, pre-emptive quality control might be a universal protective mechanism that prevents accumulation of aggregate-prone TMDs and safeguards the secretome.

## STAR★Methods

### Key Resources Table

**Table:** 

REAGENT or RESOURCE	SOURCE	IDENTIFIER
**Antibodies**		

Ani-HA, mouse monoclonal	Biolegend	Cat# MMS-101R
Anti-Sec22, rabbit polyclonal	Miller Lab	N/A
Anti-Gap1, rabbit polyclonal	Schekman Lab	N/A
Anti- Sec61, rabbit polyclonal	Schekman Lab	N/A
p-eIF2a (S52), rabbit polyclonal	Invitrogen	Cat# 44-728G

**Chemicals, Peptides, and Recombinant Proteins**		

Cycloheximide	Sigma Aldrich	Cat# C7698
Protein A Sepharose 4 Fast Flow	GE Healthcare	Cat# 17-5280-01
Protein G Sepharose 4 Fast Flow	GE Healthcare	Cat# 17-0618-01
Oligomycin	Generon	Cat# A5588
RNase OUT recombinant inhibitor	Invitrogen	Cat# 10777019
TRIzol™ Reagent	Invitrogen	Cat# 15596026
Trypsin	Sigma Aldrich	Cat# T9935
Trypsin inhibitor	Sigma Aldrich	Cat# T9003
EasyTag™ EXPRESS^35^S Protein Labeling Mix	Perkin Elmer	Cat# NEG772002MC
TRAN35S-LABEL, Metabolic Labeling Reagent	MP Biomedicals™	Cat# MP015100614 (discontinued)

**Critical Commercial Assays**		

iScript cDNA synthesis kit	BioRad	Cat# 1708891
KAPA Sybr fast universal kit	Sigma Aldrich	Cat# KK4601
PureLink Dnase	Invitrogen	Cat# 12185010
PureLink RNA Minikit	Invitrogen	Cat# 12183025
QuikChange Lightning Site-Directed Mutagenesis Kit	Agilent	Cat# 210519

**Experimental Models: Organisms/Strains**		

*Mat-a deletion collection*	Dharmacon	Cat#YSC1053
*MATa his3Δ1, leu2 Δ0, met15Δ0, ura3Δ0, yor1::KANMX*	Open biosystems	LMY094
*MATα can1::STE2pr-Sp_His5, his3Δ1, leu2Δ0, met15Δ0, ura3Δ0, lypΔ1, yor1Δ::NATMX*	Miller lab	RLY122
*MATa can1::STE2pr-Sp_His5, his3Δ1, leu2Δ0, ura3Δ0, lypΔ1, yor1::NATMX, emc7::KANMX*	This paper	RLY23
*MATa can1::STE2pr-Sp_His5, his3Δ1, leu2Δ0, ura3Δ0, lypΔ1, yor1::NATMX, emc1::KANMX*	This paper	RLY25
*MATa can1::STE2pr-Sp_His5, his3Δ1, leu2Δ0, ura3Δ0, lypΔ1, yor1::NATMX, emc2::KANMX*	This paper	RLY26
*MATa can1::STE2pr-Sp_His5, his3Δ1, leu2Δ0, ura3Δ0, lypΔ1, yor1::NATMX, emc3::KANMX*	This paper	RLY27
*MATa can1::STE2pr-Sp_His5, his3Δ1, leu2Δ0, ura3Δ0, lypΔ1, yor1::NATMX, emc4::KANMX*	This paper	RLY28
*MATa can1::STE2pr-Sp_His5, his3Δ1, leu2Δ0, ura3Δ0, lypΔ1, yor1::NATMX, emc5::KANMX*	This paper	RLY29
*MATa can1::STE2pr-Sp_His5, his3Δ1, leu2Δ0, ura3Δ0, lypΔ1, yor1::NATMX, emc16::KANMX*	This paper	RLY30
*MATa can1::STE2pr-Sp_His5, his3Δ1, leu2Δ0, ura3Δ0, lypΔ1, yor1::NATMX, emc6::KANMX, emc2::LEU2*	This paper	RLY71
*MATa can1::STE2pr-Sp_His5, his3Δ1, leu2Δ0, ura3Δ0, lypΔ1, yor1::NATMX, rpl12a::KANMX*	This paper	RLY35
*MATa can1::STE2pr-Sp_His5, his3Δ1, leu2Δ0, ura3Δ0, lypΔ1, yor1::NATMX, rrp6::KANMX*	This paper	RLY13
*MATa can1::STE2pr-Sp_His5, his3Δ1, leu2Δ0, ura3Δ0, lypΔ1, yor1::NATMX, emc7::KANMX, rrp6::LEU2*	This paper	RLY187
*MATa can1::STE2pr-Sp_His5, his3Δ1, leu2Δ0, ura3Δ0, lypΔ1, yor1::NATMX, emc7::KANMX, rpl12a:LEU2*	This paper	RLY90
*MATa can1::STE2pr-Sp_His5, his3Δ1, leu2Δ0, ura3Δ0, lypΔ1, yor1::NATMX, emc7::KANMX, ltn1::LEU2*	This paper	RLY1
*MATa can1::STE2pr-Sp_His5, his3Δ1, leu2Δ0, ura3Δ0, lypΔ1, yor1::NATMX, emc7::KANMX, cue1::LEU2*	This paper	RLY93
*MATa can1::STE2pr-Sp_His5, his3Δ1, leu2Δ0, ura3Δ0, lypΔ1, yor1::NATMX, emc7::KANMX, rpn4::LEU2*	This paper	RLY84
*MATa can1::STE2pr-Sp_His5, his3Δ1, leu2Δ0, ura3Δ0, lypΔ1, yor1::NATMX, emc7::KANMX, rps0::LEU2*	This paper	RLY119
*MATa can1::STE2pr-Sp_His5, his3Δ1, leu2Δ0, ura3Δ0, lypΔ1, yor1::NATMX, emc7::KANMX, rps9::LEU2*	This paper	RLY120
*MATa his3Δ1, leu2 Δ0, met15Δ0, ura3Δ0, opi1::KANMX yor1::NATMX*	This paper	YBP171
*MATa his3Δ1, leu2 Δ0, met15Δ0, ura3Δ0, opi1::KANMX yor1::NATMX emc7::HPHMX*	This paper	YBP172
*MATα can1::STE2pr-Sp_His5, his3Δ1, leu2Δ0, met15Δ0, ura3Δ0, lypΔ1, yor1Δ::NATMX emc7::HPH*	This paper	YBP131
*MATa trp1-1, ade2, leu 2-3,112, ura3, his3-11 ssh1::KANMX4 sec61::HIS3*	[53]	RGY400
*MATa trp1-1, ade2, leu 2-3,112, ura3, his3-11 ssh1::KANMX4 sec61::HIS3 emc7::NATMX*	This paper	YBP225
*MATa trp1-1, ade2, leu 2-3,112, ura3, his3-11 ssh1::KANMX4 sec61::HIS3 emc7::NATMX hel2::TRP*	This paper	YBP197
W303-1a background *rps20Δ::NATMX4, p414GPDp-RPS20-CYC1t*	[29]	N/A
W303-1a background *rps20Δ::NATMX4, p414GPDp-RPS20-CYC1t emc7::LEU2*	This paper	YBP218
W303-1a background *rps20Δ::NATMX4, p414GPDp-rps20 K6R K8R-CYC1t*	[29]	N/A
W303-1a background *rps20Δ::NATMX4, p414GPDp-rps20 K6R K8R-CYC1t emc7::LEU2*	This paper	YBP217
W303-1a background *rps3Δ::NATMX4, p414GPDp-RPS3-CYC1t*	[29]	N/A
W303-1a background *rps3Δ::NATMX4, p414GPDp-RPS3-CYC1t emc7::LEU2*	This paper	YBP220
W303-1a background *rps3Δ::NATMX4, p414GPDp-rps3 K212R-CYC1t*	[29]	N/A
W303-1a background *rps3Δ::NATMX4, p414GPDp-rps3 K212R-CYC1t emc7::LEU2*	This paper	YBP222
*MATa can1::STE2pr-Sp_His5, his3Δ1, leu2Δ0, ura3Δ0, lypΔ1, yor1::NATMX, rrp6::KANMX eap1::LEU2*	This paper	YIB01
*MATα can1::STE2pr-Sp_His5, his3Δ1, leu2Δ0, met15Δ0, ura3Δ0, lypΔ1, yor1Δ::NATMX, SEC63-sfGFP::HIS3*	This paper	NGY528
*MATa, leu2 Δ0, met15Δ0, ura3Δ0, yor1::NATMX, emc7::HPHMX, SEC63-sfGFP::HIS3*	This paper	NGY529
*MATa, leu2 Δ0, met15Δ0, ura3Δ0, yor1::NATMX, opi1::KANMX, SEC63-sfGFP::HIS3*	This paper	NGY530
*MATa, leu2 Δ0, met15Δ0, ura3Δ0, yor1::NATMX, opi1::KANMX, emc7::HPHMX, SEC63-sfGFP::HIS3*	This paper	NGY531
*MATa can1::STE2pr-Sp_His5, his3Δ1, leu2Δ0, ura3Δ0, lypΔ1, yor1::NATMX, emc7::KANMX, hel2::LEU2*	This paper	YIB037
*MATa can1::STE2pr-Sp_His5, his3Δ1, leu2Δ0, ura3Δ0, lypΔ1, yor1::NATMX, emc7::KANMX, dom34::LEU2*	This paper	YIB038
*MATa can1::STE2pr-Sp_His5, his3Δ1, leu2Δ0, ura3Δ0, lypΔ1, yor1::NATMX, emc7::KANMX, slh1::LEU2*	This paper	YIB039

**Oligonucleotides**		

AATTATGGGATGCATTGGTGAGAGG (qPCR Yor1 fw)	IDT	OIB204
TCACCTAAGGAGAAATTGGAGCCC (qPCR Yor1 rv)	IDT	OIB205
GGTTTGGAATCTGCCGGTATTG (qPCR Act1 fw)	IDT	OIB210
CAAAGCGGTGATTTCCTTTTGC (qPCR Act1 rv)	IDT	OIB211

**Recombinant DNA**		

pRS316(URA) *YOR1-HA*	[12]	pEAE83
ΔF670 and R1116T mutations in pEAE83	[14]	LMB287
G278R (Yor1 G278R) mutation in pEAE83	[18]	spQC35
R387G (Yor1R387G) mutation in pEAE83	[19]	spQC36
I1084P (Yor1 I1084P) mutation in pEAE83	[18]	spQC39
F270S and R1168M mutations in LMB287	[18]	JH079
R1116T (Yor1 I1084P, R1116T) mutation in spQC39	This paper	ICL4RT
Δ71AE73A (Yor1D71A, E73A) mutation in pEAE83	[13]	pLM31
pRS315(LEU) *SEC61*	[34]	pBW11
pRS315(LEU) *sec61-R275E/R406E*	[53]	N/A
pRS315(LEU) *sec61-N302L*	[34]	pEM634
pRS315(LEU) *sec61-Q129N*	[34]	pEM629
pRS315(LEU) *sec61-R275E/K464E/K470E*	[33]	pEM905
pRS316(URA) YOR1-GFP	[12]	pEAE93
pRS316(URA) Yor1-DF670-GFP	[13]	LMB037
pRS316(URA) Ste6-166-HA	[54]	pSM1083
Yep(URA) Ycf1DF713-GFP	Susan Michaelis	pSM1755
pRS316(URA) CPY^∗^-HA	Peter Walter	pCP258
pFA6-LEU	[55]	LMB138
pFA6a-KanMX6	[55]	LMB298
pFA6a-GFP(S65T)-HIS3MX6	[55]	LMB303

**Software and Algorithms**		

Nikon NIS Elements software	Nikon	RRID: SCR_014329
Andor iQ3 software	Oxford Instruments	RRID: SCR_014461
ImageQuant software	GE Healthcare	RRID: SCR_014246
Prism v.8	GraphPad	RRID: SCR_002798
ImageJ (FiJi)	NIH	RRID: SCR_002285
QuantStudio Real-Time PCR Software v1.3	Thermo Fisher	https://www.thermofisher.com/uk/en/home/life-science/pcr/real-time-pcr/real-time-pcr-instruments/quantstudio-qpcr-product-portfolio.html

### Lead Contact and Materials Availability

Further information and requests for resources and reagents should be directed to and will be fulfilled by the Lead Contact, Elizabeth Miller (emiller@mrc-lmb.cam.ac.uk). Yeast strains and plasmids generated in this study have not been deposited in an external repository but are available for distribution on request from the Lead Contact. All data are available in the main text or the supplementary materials. All materials will be available to any researcher for purposes of reproducing or extending our findings.

### Experimental Model and Subject Details

*Saccharomyces cerevisiae* strains used in the study are listed in the Key Resources Table. Cultures were grown at 30°C in standard rich medium (YPD: 1% yeast extract, 2% peptone, and 2% glucose) or synthetic complete medium (SC: 0.67% yeast nitrogen base and 2% glucose supplemented with amino acids as needed).

### Method Details

#### Strain construction

The query strain (RLY122) used to make double mutants was made by Silvere Pagant using PCR-mediated homologous recombination to knock out the *YOR1* gene (MATα *can1::STE2pr-Sp_His5, his3Δ1, leu2Δ0, met15Δ0, ura3Δ0, lypΔ1, yor1Δ::NATMX). YOR1/EMC* double mutants and various *YOR1/EMC7/xxx* triple mutants were created by the synthetic genetic array (SGA) method [56]. The query strain was mated to different deletion mutant strains from the MATa deletion collection (Dharmacon). Strains were mated on YPD plates overnight, followed by diploid selection on YPD +G418 +NAT. Strains were then sporulated for a week at 25°C before two rounds of haploid double/triple mutant selection [56]. The resulting strains were then transformed with different *YOR1* constructs for phenotypic analysis and labeling experiments. The *sec61Δ ssh1Δ emc7Δ* triple mutant strain was made by crossing and tetrad dissection to avoid suppression effects, then transformed with various Yor1 plasmids for labeling experiments. Additional mutants were made by PCR-mediated homologous recombination using Longtine cassettes with 40 bp homology arms immediately upstream and downstream of the insertion [55].

#### Plasmids

The plasmids used in this study are listed in the Key Resources Table. pEAE83 bearing *YOR1-HA* in pRS316 was a gift from Scott Moye-Rowley (University of Iowa). This plasmid was the basis for site-directed mutagenesis by using QuikChange mutagenesis (Stratagene, La Jolla, CA) to obtain various hemagglutinin (HA)-tagged Yor1 mutants [18]. To facilitate genetic screening approaches, we use a gain-of-function allele, R_1116_T, which confers increased drug clearance activity to both wild-type and misfolded forms of the protein, yet doesn’t impact folding or biogenesis. In this and our previous study [14], we use Yor1-ΔF_670_/R_1116_T for both phenotypic and biochemical analyses, but refer to the protein as Yor1-ΔF for simplicity.

#### Oligomycin sensitivity assay

Strains were grown to saturation in SC -ura medium overnight at 30°C. 10-fold serial dilutions were made in 96 well plates before spotting onto YPEG (1% yeast extract, 2% peptone, 3% ethanol, 3% glycerol) plates containing different concentrations of oligomycin (0, 0.2, 0.6 μg/mL). Plates were scanned at day 4 or day 5 after spotting and growth at 30°C.

#### Metabolic labeling

Cells were grown to mid-log phase (A600 ∼0.5) in complete synthetic medium; a total of 8 A600 cells were harvested, washed and resuspended in SC medium (2ml) lacking Met/Cys, and incubated at 30°C for 15 min while gently shaking. Nascent proteins were labeled at 30°C for different times (2.5, 5, 10, 15 min) by adding 30 μCi of 35S-Met/Cys (MP Biomedicals or Perkin Elmer)/A600 cells (24 μL of label/reaction). 500 μL of cells were harvested per strain at each time point using a pre-set timer and were transferred to chilled tubes containing a final concentration of 20mM NaN_3_. Cells were washed once with 20mM NaN_3_ and resuspended in 100 μL of 1% SDS. Cells were disrupted by glass bead lysis (15 min, 4°C), heated to 55°C for 5 min, and centrifuged at low speed (500 g) for 30 s. Lysates were diluted with 5 volumes of immunoprecipitation buffer (50 mM Tris, pH 7.5, 160mM NaCl, 1% Triton X-100, and 2mM NaN_3_), and cleared by centrifugation at 13,000 rpm for 5 min. Proteins were immunoprecipitated using monoclonal anti-HA antibodies, precoupled to protein G-Sepharose beads (GE Healthcare), or polyclonal antibodies against Sec22 or Gap1 precoupled to protein A-Sepharose beads (GE Healthcare). Immune complexes were separated by SDS-PAGE and analyzed by phosphorimaging analysis using a Typhoon PhosphorImager (GE Healthcare). Incorporation were quantified using ImageQuant (GE Healthcare) or FiJi (NIH) software.

#### Protein aggregation assay

Wild-type and *emc7Δ* strains were grown to mid-log phase in complete synthetic medium; cells were harvested, washed and resuspended in SC medium lacking Met/Cys, and incubated at 30°C for 15 min while gently shaking. Nascent proteins were labeled at 30°C for 10 min with 30 μCi of TRAN35S-Label (MP Biomedicals)/A600 cells. For separation into soluble and insoluble fractions, cells were harvested and washed with 20mM NaN_3_ and incubated on ice for 5 min. Spheroplasts were prepared by treating cells initially with an alkaline buffer (100mM Tris pH9.4, 10mM DTT, 100mM NaN_3_) for 10 min at room temperature followed by treatment with lyticase in spheroplasting buffer (10mM Tris pH7.4, 0.7M sorbitol, 1mM DTT, 20mM NaN_3_) for 25 min at 30°C. Spheroplasts were then collected, resuspended in 50μL hypo-osmotic lysis buffer (20mM HEPES pH6.8, 0.4M sorbitol, 150mM KOAc, 2mM Mg (OAc)_2_, 0.5mM EGTA) and frozen at −80°C. Frozen cell lysates were thawed, and subjected to centrifugation (15,000 rpm, 5 min. 4°C). The supernatant corresponding to the soluble fraction was removed and mixed with an equal volume of 2X SDS sample buffer, and the insoluble pellet was resuspended in 100μL of 1X SDS sample buffer before loading on SDS-PAGE and PhosphorImage analysis as described above.

#### Limited Proteolysis

Cells were grown to mid-log phase in complete synthetic medium; a total of 5 A600 cells were harvested, washed and resuspended in SC medium lacking Met/Cys, and incubated at 30°C for 15 min while gently shaking. Cells were metabolically labeled at 30°C for 10 min with 60 μCi of TRAN35S-Label (MP Biomedicals)/A600 cells. Cells were harvested and washed with 20mM NaN_3_ and incubated on ice for 5 min. Spheroplasts were prepared by treating cells initially with an alkaline buffer (100mM Tris pH9.4, 10mM DTT, 100mM NaN_3_) for 10 min at room temperature followed by treatment with lyticase in spheroplasting buffer (10mM Tris pH7.4, 0.7M sorbitol, 1mM DTT, 20mM NaN_3_) for 25 min at 30°C. Spheroplasts were then washed in lysis buffer (20mM HEPES pH6.8, 0.4M sorbitol, 150mM KOAc, 2mM Mg (OAc)_2_, 0.5mM EGTA) and were frozen at −80°C. The following day, spheroplasts were thawed on ice and washed in low-acetate B88 buffer (20mM HEPES pH 6.8, 250mM sorbitol, 50mM KOAc, 5mM Mg(OAc)_2_) followed by two washes with B88 (20mM HEPES pH 6.8, 250mM sorbitol, 150nM KOAc, 5mM Mg(OAc)_2_). Lysed cells were resuspended in 100 μL B88 buffer and split into four 25 μL reactions per strain. Each reaction was treated with a final concentration of 0, 25, 50, 100 ng/μl trypsin for 10 min on ice. Digestion was terminated by addition of soybean trypsin inhibitor to all reactions followed by incubation on ice for 15 min and by two washes with B88. After solubilization with 1% SDS, spheroplasts were disrupted by glass bead lysis and heated at 55°C for 5 min. The resulting protein extracts were diluted with 5 volumes of immunoprecipitation buffer (50 mM Tris, pH 7.5, 160mM NaCl, 1% Triton X-100, and 2mM NaN_3_), and cleared by centrifugation. Yor1 fragments were immunoprecipitated from the cleared supernatant and analyzed by SDS-PAGE and PhosphorImage analysis as described above.

#### GFP Imaging

Imaging was performed on cells grown to mid-log phase at 30°C in selective media. Images were taken on a Nikon TE2000 inverted fluorescence microscope with a 100x/1.49NA oil immersion objective and an sCMOS camera and collected using the Nikon NIS elements software. For imaging of cortical ER in the *opi1Δ* strains, images were taken on an Andor Revolution Spinning Disk microscope with a 40x/1.3NA oil immersion objective and an EMCCD camera. Images from the mid and cortical sections of cells were collected using Andor iQ3 software.

#### qPCR

Yeast cells in mid-log phase were collected and RNA extracted using PureLink RNA Minikit (Thermo Fisher) with Trizol reagent and on-column DNase treatment according to the manufacturer’s protocol. Eluted RNA was used to prepare cDNA using iScript (BioRad) kits, according to the manufacturer’s protocol. qPCR was performed using KAPA Sybr Fast Universal kits on a Viia 7 Real Time PCR system, with primers that amplify *YOR1* and *ACT1* in triplicate, along with appropriate controls. For each condition, three biological replicates were performed. *YOR1* was quantified relative to *ACT1* using a relative standard curve. The standard curve was generated from pooled cDNA to make a highest standard concentration, with 100 as the assigned quantity. Five more standards were then generated with a 5 fold dilution series. Each cDNA sample to be measured was diluted 10-fold to ensure transcript abundance was within the range of the standard curve. The Ct from each sample was measured against the standard curve to find the assigned quantity, and then the assigned quantity of *YOR1* was divided by that of *ACT1* to calculate the relative abundance, using the QuantStudio Real Time PCR software (ThermoFisher).

#### Polysome profiling

Yeast cells in mid-log phase (OD600 0.5-0.6) were treated with 100ug/mL cycloheximide for 15 min, then harvested and resuspended in 400μl lysis buffer (20mM HEPES 7.4, 5mM Mg(CH_3_COO)_2,_ 50mM KCl, 100ug/mL cycloheximide) supplemented with 20μl RNaseOUT (Thermo Fisher). Cells were disrupted by glass bead lysis for 10mins at 2000rpm and 4°C, then the lysate was cleared by centrifugation for 1 min at 3200rpm and 4°C, followed by 10 min at 13000rpm and 4°C. Extracts were frozen in liquid nitrogen and stored at −80°C until use. 8 A_260nm_ units of the lysate were loaded onto a linear 5% - 45% (w/v) sucrose gradient in polypropylene 14 × 95mm centrifuge tubes and centrifuged for 2.5 h at 284,600 x g and 4°C. Gradients were fractionated using an ÄKTAprime plus liquid chromatography system and a Brandel gradient fractionator with continuous UV monitoring (A_254nm_). The collected fractions were frozen in liquid nitrogen and stored at −80°C.

### Quantification and Statistical Analyses

For radiolabeling experiments, band intensity was measured using a Typhoon PhosphorImager (GE Healthcare) and quantified using either ImageQuant (GE Helthcare) or ImageJ/FiJi (NIH) software. Statistical analyses were performed using GraphPad Prism software. Plots were also generated using this software. Details of statistical tests for individual experiments can be found in the figure legends. In general, n refers to independent biological replicates of a given experiment. Significance was defined as a p value less than 0.05.

### Data and Code Availability

Translation efficiency was calculated from published data [38]. TE for each gene with a minimum of 5 rpkm was calculated as the ratio of ribosome-protected footprints (RPF) to RNA-seq rpkm. Secretome proteins were identified and classified/sorted using annotated datasets [39, 40]. Protein abundance was downloaded from pax-db.org. Data generated for this analysis is available from the Lead Contact.

## Supplemental Information

Document S1. Figures S1–S6

Document S2. Article plus Supplemental Information
