# Supplementary material for: Pre-emptive Quality Control of a Misfolded Membrane Protein by Ribosome-Driven Effects
Source: Curr Biol. 2020 Mar 9;30(5):854–864.e5. doi: 10.1016/j.cub.2019.12.060 (PMC7063571; doi:10.1016/j.cub.2019.12.060)
Supplement: Document S1. Figures S1–S6 [file mmc1.pdf]

Current Biology, Volume *30*

Supplemental Information

Pre-emptive Quality Control of a Misfolded Membrane Protein by Ribosome-Driven Effects

Ramya Lakshminarayan, Ben P. Phillips, Imogen L. Binnian, Natalia Gomez-Navarro, Norberto Escudero-Urquijo, Alan J. Warren, and Elizabeth A. Miller

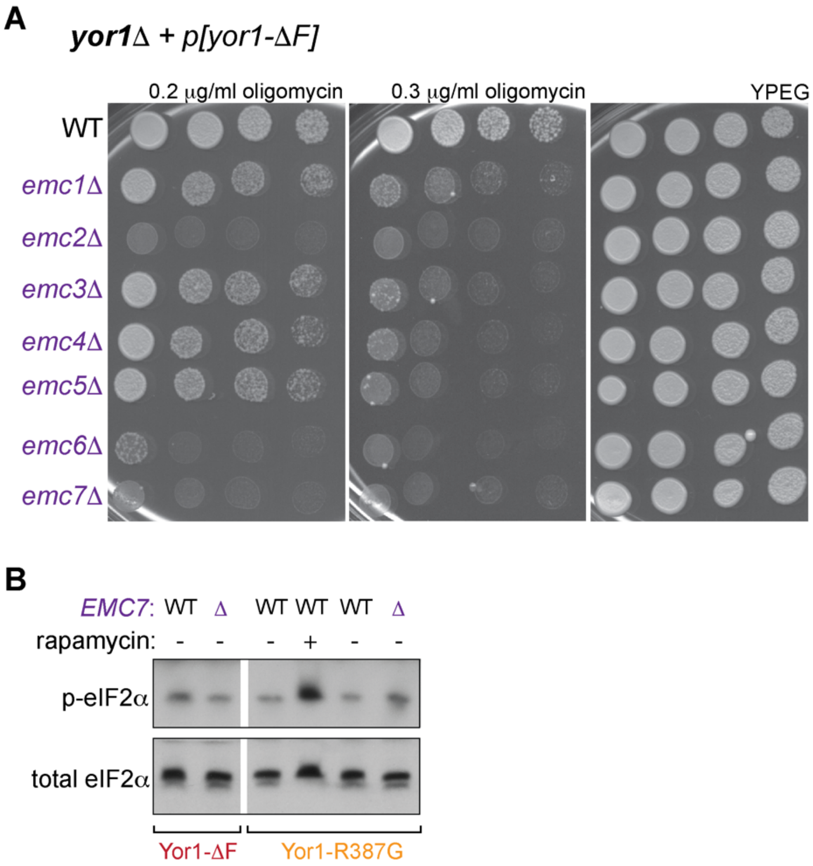

Figure S1. Loss of EMC reduces Yor1-ΔF function. Related to Figure 1. (A) Yeast strains deleted for *YOR1* and the additional genes indicated were transformed with a plasmid expressing Yor1-ΔF(HA) and grown overnight in SD -ura before serial dilution and spotting onto YPEG media (right panel) or YPEG supplemented with the oligomycin as indicated. Cells were incubated at 30ºC for 3-4 days and plates imaged. Mutation of any of the EMC components reduced the oligomycin resistance associated with Yor1-ΔF expression. **(B)** Lysates from WT and *emc7Δ* strains expressing Yor1-ΔF or the functional ICL2 mutant (Yor1-R_{387}G) were probed with antibodies against eIF2α and phosphor-eIF2α. Neither condition yielded increased phosphorylation indicative of ISR activation. In contrast, pre-treatment of WT cells with rapamycin clearly induced eIF2α phosphorylation.

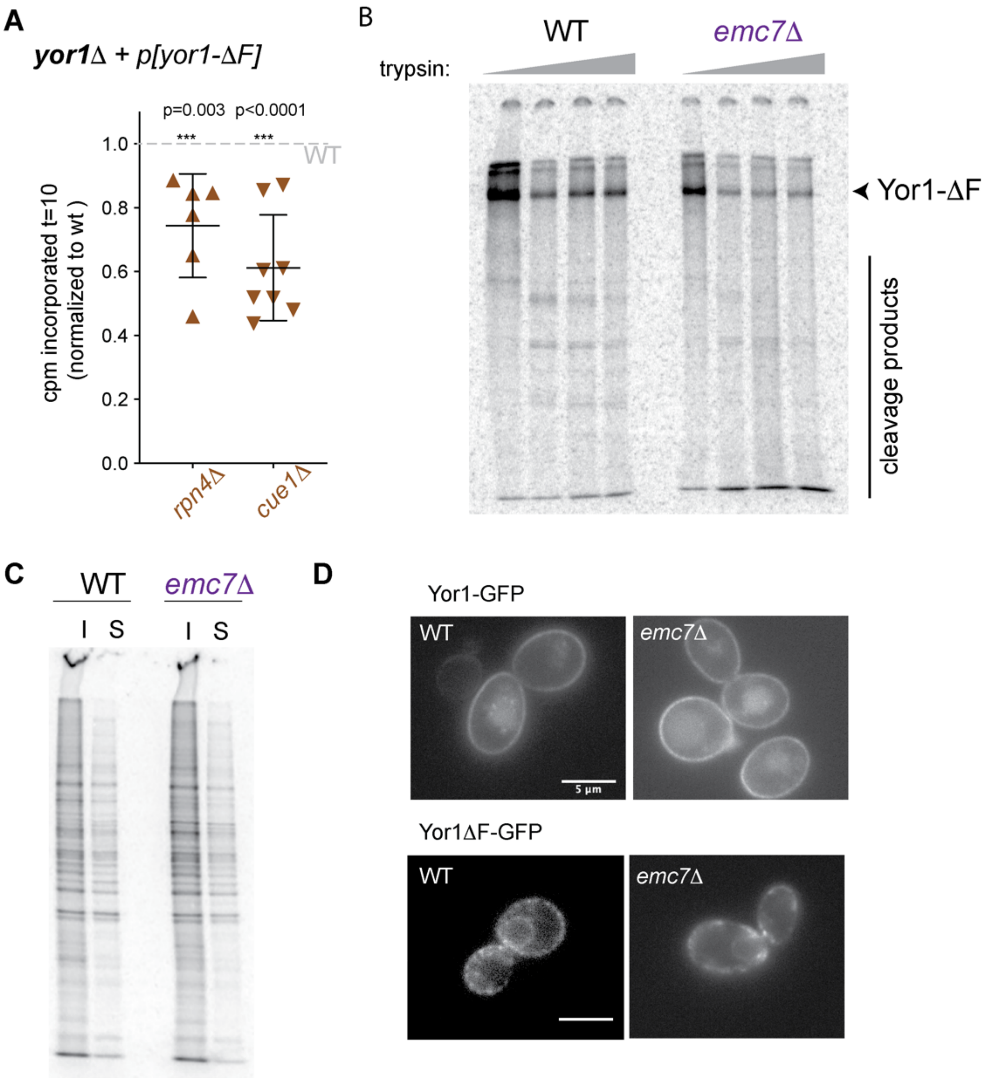

Figure S2. EMC loss doesn't exacerbate ERAD, aggregation or misfolding. Related to Figure 3. **(A)** Strains deleted for *YOR1* and either *RPN4* or *CUE1* were metabolically labeled and incorporation at t=10 relative to a WT strain was quantified. Both mutants showed reduced incorporation of Yor1-ΔF relative to WT. Statistical analysis was an unpaired Student's t-test; error bars depict SD. **(B)** Radiolabeled cells were spheroplasted, gently lysed and subject to limited proteolysis before immunoprecipitation. No major differences in cleavage patterns were detected between WT and *emc7Δ* cells. **(C)** WT and *emc7Δ* cells subjected to metabolic labeling were spheroplasted and separated into insoluble (I) and soluble (S) fractions prior to SDS-PAGE and PhosphorImage analysis. No major differences in the soluble and insoluble fractions were apparent. **(D)** Yor1-GFP and Yor1-ΔF-GFP were expressed in WT and *emc7Δ* cells. No differences in intracellular puncta/aggregates were detected in the *emc7Δ* strain. Conversely, expression of Yor1-ΔF-GFP in a severe Sec61 targeting mutant resulted in strong punctate accumulation (see Figure 6B, middle panel). Scale bar is 5 µm.

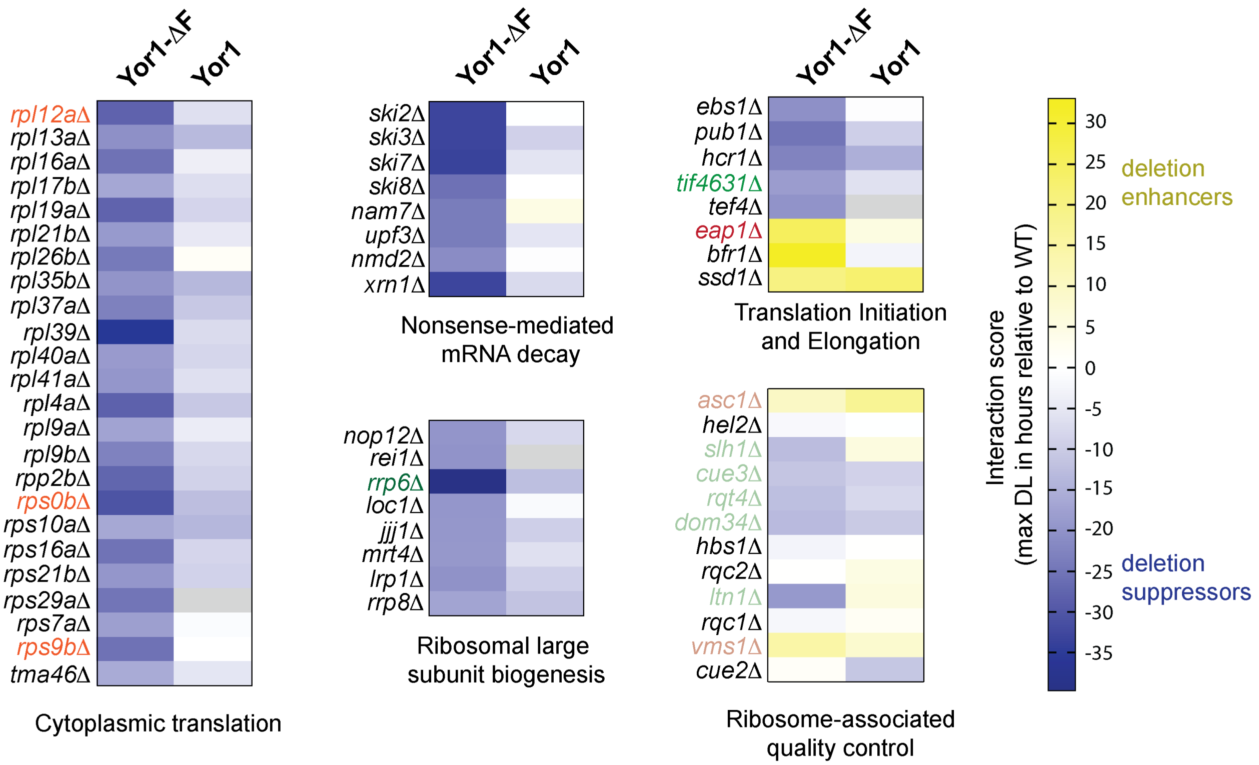

Figure S3. Genetic signatures of pre-emptive quality control: Translation-related proteins influence Yor1-ΔF function. **Related to Figures 3 and 5.**

Heat map of interaction scores from a phenotypic screen for factors that influence Yor1-ΔF biogenesis [14]. The interaction score represents the change in lag-phase growth (L, the time to half-maximal growth measured in hours) of a mutant strain relative to the wild-type control on a given concentration of oligomycin. A negative value corresponds to improved growth relative to WT (ie. less lag and therefore faster growth). Darker blue corresponds to better growth (ie. stronger suppression of oligomycin sensitivity associated with Yor1-ΔF expression). Interaction scores for various classes of GO-enriched functional terms are shown, alongside the corresponding interaction scores for equivalent strains expressing WT Yor1. Additional hits relevant to Translation Initiation/Elongation and Ribosome-associated Quality Control are also included.

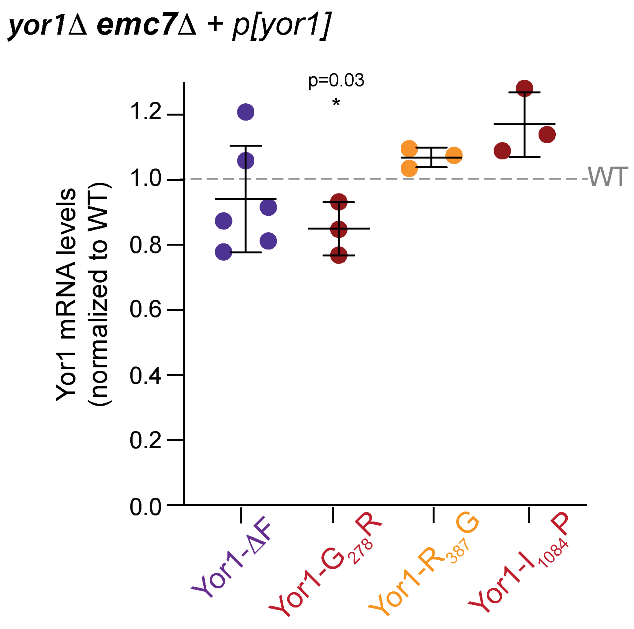

Figure S4. mRNA levels of Yor1 mutants in *emc7Δ* cells. Related to Figure 3.

Steady state mRNA levels for the indicated *YOR1* alleles were quantified by qPCR in WT and *emc7Δ* cells. CT values for each test sample were compared to a standard curve, then normalized to actin similarly measured according to a standard curve, and the relative levels in *emc7Δ* cells depicted relative to WT. Each biological replicate (individual data points) was analyzed in triplicate technical replicates; Statistical test was an unpaired Student's t-test; error bars are SD.

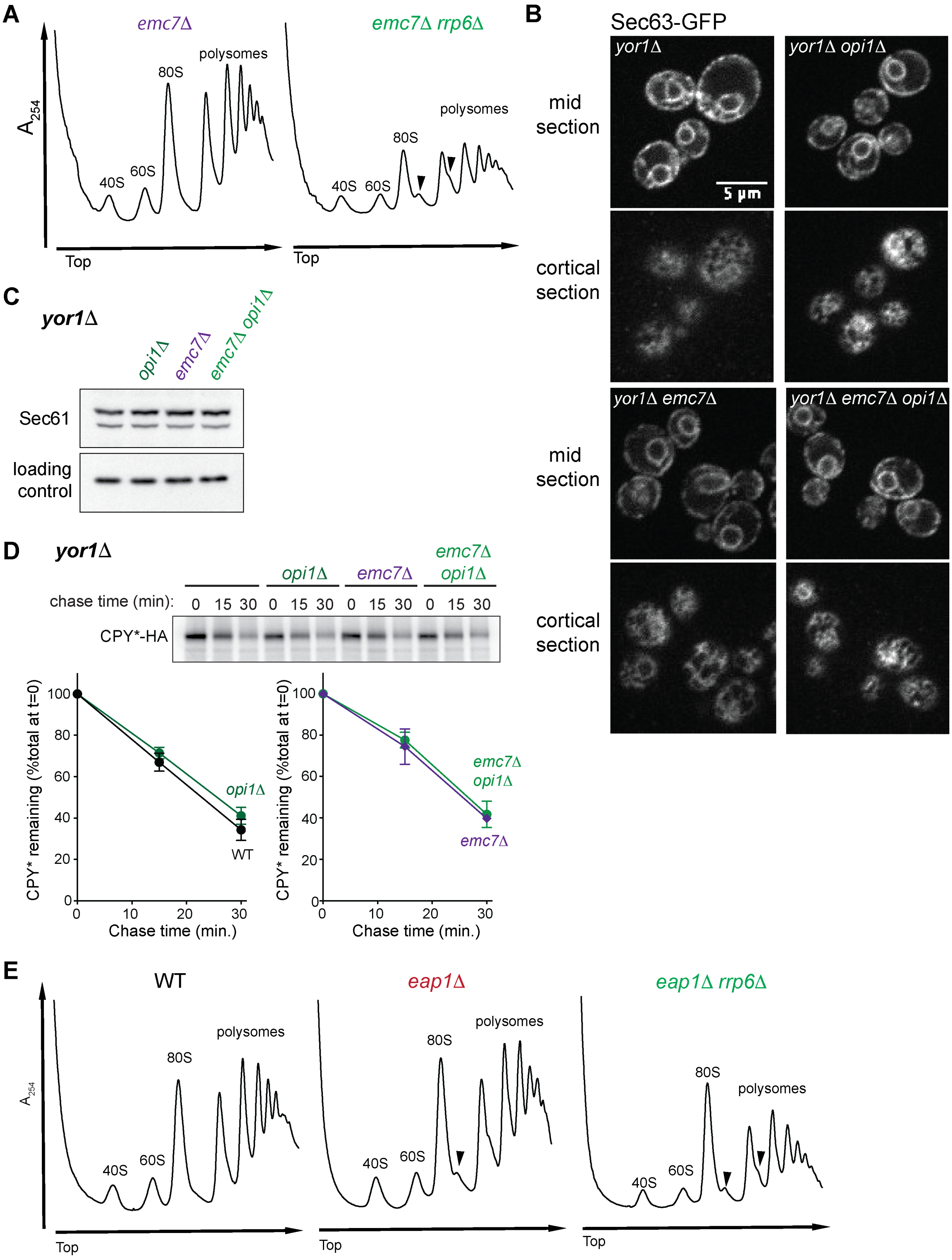

A
emc7Δ
emc7Δ rrp6Δ
A254
40S
60S
80S
polysomes
Top
B
Sec63-GFP
yor1Δ
yor1Δ opi1Δ
yor1Δ emc7Δ
yor1Δ emc7Δ opi1Δ
mid section
cortical section
5 μm
C
yor1Δ
opi1Δ
emc7Δ
emc7Δ opi1Δ
Sec61
loading control
D
yor1Δ
chase time (min):
0 15 30
CPY*-HA
CPY* remaining (%total at t=0)
Chase time (min.)
opi1Δ
WT
emc7Δ opi1Δ
emc7Δ
E
WT
eap1Δ
eap1Δ rrp6Δ

Figure S5. Ribosome dilution effects influence synthesis defects. Related to Figure 5.

(A) Representative polysome profiles of lysates prepared from the indicated strains reveals that the *rrp6Δ* mutant contains reduced abundance of 60S, 80S and polysomes, as well as the appearance of "halfmers", indicated by arrowheads. **(B)** ER morphology was examined using Sec63-sfGFP integrated into the genomic locus in the strains indicated. Confocal imaging of mid-cell and cortical planes revealed more abundant and sheet-like ER in the cortical ER in strains deleted for *OPI1*, as described previously [36]. **(C)** Steady state levels of Sec61 in the strains indicated were measured from whole-cell lysates by immunoblotting using a Sec61-specific polyclonal antibody. No change in abundance was observed upon ER proliferation in the *opi1Δ* mutants. **(D)** Pulse-chase experiments of the model misfolded protein, CPY*-HA in the strains indicated revealed no change in ERAD associated with ER expansion upon loss of *OPI1*. n=3; error bars represent SD. **(E)** Representative polysome profiles prepared from lysates of the indicated strains shows no major differences upon loss of *EAP1*, but reduced ribosome abundance upon additional loss of *RRP6*.

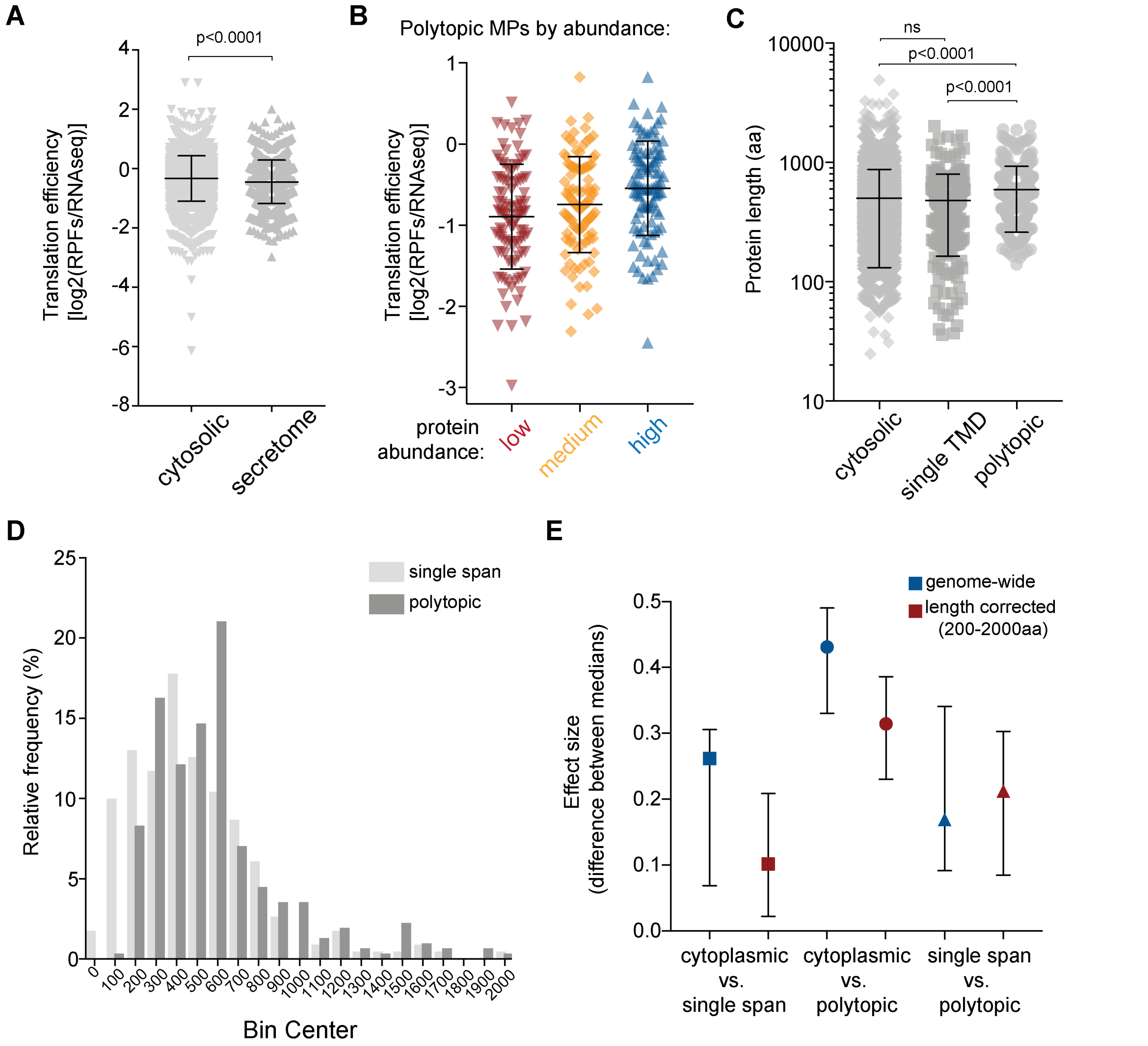

Figure S6. Secreted proteins have reduced translation efficiency. Related to Figure 6.

(A) Secretome proteins had significantly lower translation efficiency (TE) than cytosolic proteins. **(B)** Polytopic membrane proteins (>4 TMDs) were separated into low-, medium- and high-abundance classes based on PaxDB data. Higher-abundance proteins had higher TE. **(C)** Polypeptide length is plotted for different protein classes; polytopic membrane proteins (>4 TMDs) tend to be longer than cytosolic or single-pass TM proteins. **(D)** Length distribution of single-pass and polytopic membrane proteins were plotted to find a length window appropriate for comparison of length-controlled proteins. **(E)** Effect size was measured for the different comparisons indicated, either considering the entire proteome, or the length-controlled population. Statistical tests were all Mann-Whitney U tests and error bars represent SD.
